# 5-Lipoxygenase inhibition reduces inflammation and neuronal apoptosis via AKT signaling after subarachnoid hemorrhage in rats

**DOI:** 10.18632/aging.202869

**Published:** 2021-04-20

**Authors:** Liu Liu, Ping Zhang, Zhaosi Zhang, Yidan Liang, Hong Chen, Zhaohui He, Xiaochuan Sun, Zongduo Guo, Yongbing Deng

**Affiliations:** 1 Department of Neurosurgery, Chongqing Emergency Center, Chongqing University Center Hospital, School of Medicine, Chongqing University, Chongqing, China; 2 Department of Neurosurgery, The First Affiliated Hospital of Chongqing Medical University, Chongqing, China; 3 Department of Cerebrovascular Diseases, The First Affiliated Hospital of Zunyi Medical University, Guizhou, China

**Keywords:** 5-LOX, subarachnoid hemorrhage, early brain injury, inflammation, apoptosis

## Abstract

Early brain injury (EBI) is a major contributor to the high mortality and morbidity after subarachnoid hemorrhage (SAH). Inflammatory responses and neuronal apoptosis are important causes of EBI. Because 5- lipoxygenase (5-LOX) is known to be involved various central nervous system diseases, we investigated the effects of 5-LOX inhibition during EBI after SAH. Zileuton and LY294002 were used to inhibit expression of 5-LOX and Akt, respectively. We found that 5-LOX expression was significantly increased in the cytoplasm of cortical neurons after SAH and was accompanied by upregulated expression of the inflammatory factors LTB4, TNF-α, IL-1β and IL-6; upregulation of the pro-apoptotic factor Bax; downregulation of the anti-apoptotic factor Bcl-2; and an increased apoptosis rate. Gastric Zileuton administration significantly suppressed all of those effects and improved neurological function. Zileuton also upregulated activated (phosphorylated) AKT levels, and these beneficial effects of Zileuton were abolished by intracerebroventricular infusion of the PI3K inhibitor LY294002. Taken together, these findings indicate that 5-LOX mediates pro-inflammatory and pro-apoptotic effects that contribute to EBI after SAH and that those effects are suppressed by activation of PI3K/Akt signaling. This suggests targeting 5-LOX may be an effective approach to treating EBI after SAH.

## INTRODUCTION

Subarachnoid hemorrhage (SAH) is a kind of potentially lethal stroke and is associated with high rates of disability and mortality [1]. Early brain injury (EBI) is the most important factor contributing to a poor prognosis in SAH patients, which is characterized by a series of pathological derangements that occur within 72 h after the onset of SAH [2]. Previous studies have shown that both inflammation and apoptosis contribute to EBI [3, 4].

5-Lipoxygenase is a member of the family of lipoxygenase enzyme and catalyzes the conversion of arachidonic acid (AA) to the inflammatory molecule leukotriene (LT), which is an effective mediator involved in several inflammatory diseases [5]. Zileuton is a 5-LOX inhibitor, which exhibits inhibitory actions against inflammatory diseases and attenuates neuronal apoptosis with cerebral ischemia in rats [6]. Whether Zileuton attenuates inflammation and neuronal apoptosis following SAH in rats remains unclear, however. It is well documented that phosphatidylinositol-3 kinase (PI3K)-AKT involved in several cellular pathways, including cellular proliferation, survival, and apoptosis [7]. Recent research confirms that activation of the PI3K-AKT pathway was also correlated with EBI following SAH [8]. Previous studies indicate that AKT acts downstream of 5-LOX and that Zileuton can suppress the activity of PI3K [9, 10].

Against that background, we established an SAH model by intravascular puncture method and studied the effect of 5-LOX on the inflammation and apoptosis seen after SAH. We then utilized the 5-LOX inhibitor Zileuton and specific inhibitors of PI3K/AKT to suppress 5-LOX expression and PI3K/AKT signaling in order to evaluate the role of 5-LOX in the EBI after SAH.

## RESULTS

### SAH increases 5-LOX, bax, NF-κB levels and decreases Bcl2 levels

**Figure 1:**
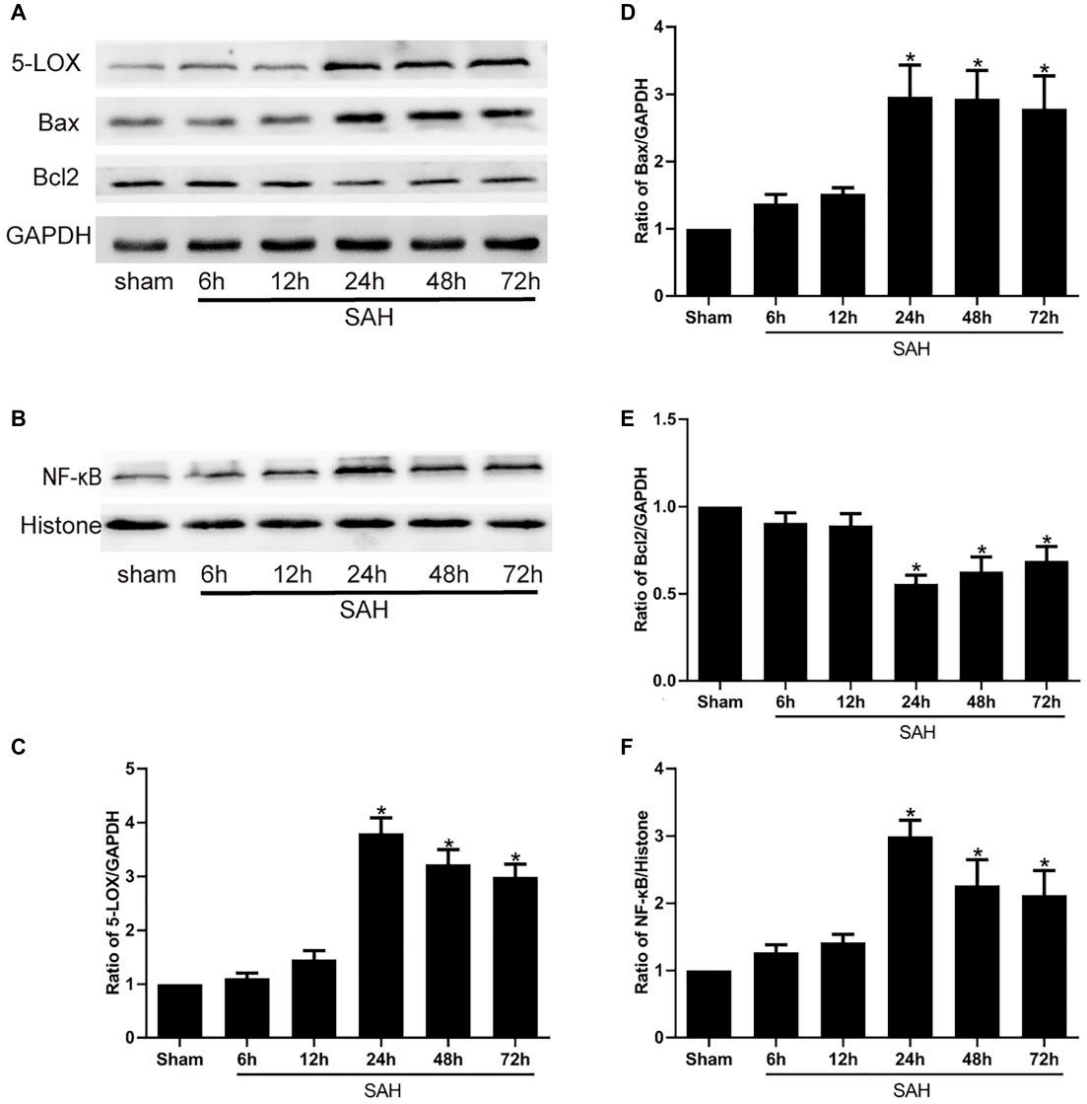
**Levels of 5-LOX and NF-κB, Bax and Bcl2 protein at the indicated times after SAH.** (**A**, **B**) Representative Western blots showing expression levels of 5-LOX, NF-κB, Bcl-2 and Bax. (**C**–**F**) Densitometric quantification of the protein bands. *N* = 6 in each group; ^*^P < 0.05 vs sham group.

We first used Western blot analyses to assess the changes in the expression of 5-LOX and various inflammatory and apoptotic factors, including NF-κB, Bax and Bcl2, at selected times after induction of SAH. The results showed that 5-LOX expression was increased and peaked at 24 h following SAH (Figure 1A, 1C). This increase in 5-LOX was accompanied by upregulation of NF-κB (Figure 1B, 1F). Bcl-2 is anti-apoptotic mediator, while Bax exerts pro-apoptotic effects. We observed that expression of Bcl-2 was decreased after SAH (Figure 1A and 1E), whereas Bax levels were increased (Figure 1A, 1D).

### Zileuton decreases expression of 5-LOX by activating the PI3K/AKT pathway

**Figure 2:**
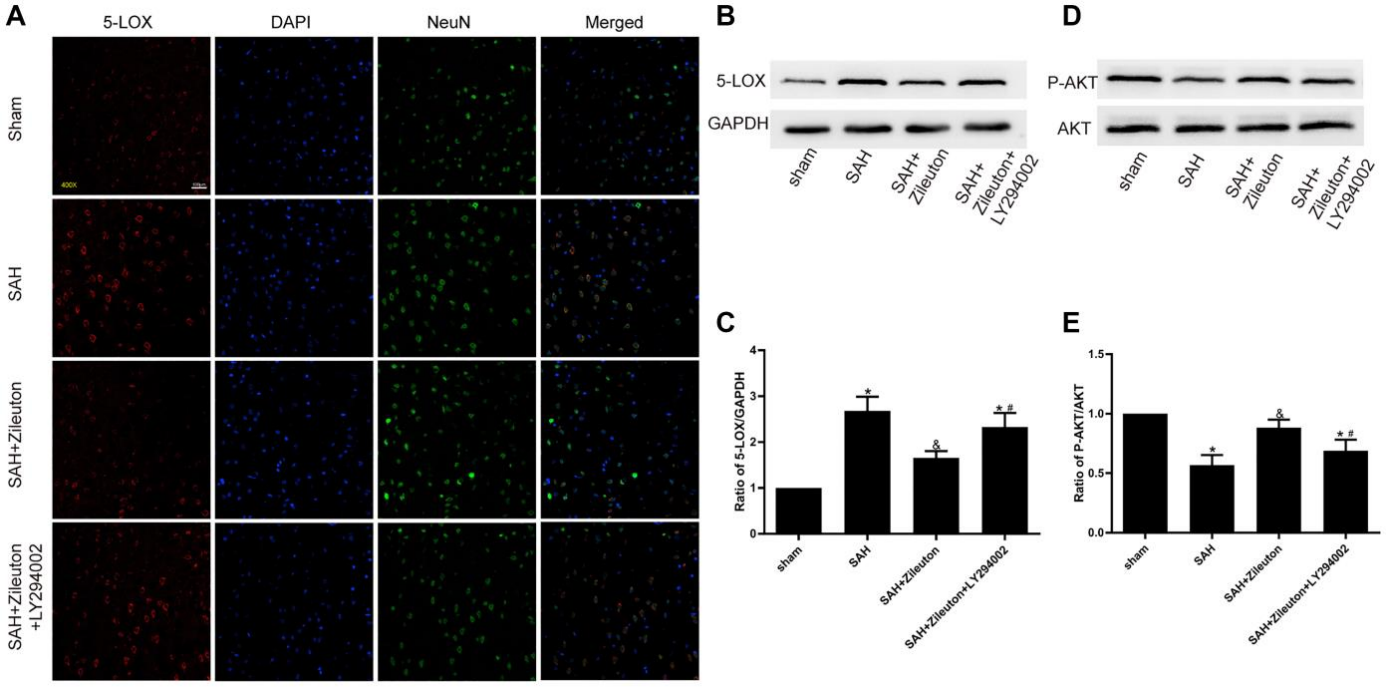
**Zileuton decreases 5-LOX expression through PI3K/AKT activation.** (**A**) Immunofluorescent staining for 5-LOX (red) and NeuN (green) in the cortex of rats in the sham, SAH, SAH + Zileuton and SAH + Zileuton + LY294002 groups. Nuclei were counterstained with DAPI (blue). Final magnification, 400×; scale bars = 100 μm, *n* = 6 per group. (**B**–**E**) Representative images and quantitative analysis of 5-LOX and p-AKT after Zileuton treatment with and without LY294002. *N* = 6 in each group, ^*^P < 0.05 vs sham group and ^&^*P* < 0.05 vs SAH group, ^#^*P* < 0.05 vs SAH + Zileuton group.

Double immunofluorescent staining of 5-LOX and the neuronal marker NeuN revealed that after SAH 5-LOX was widely expressed in NEUN-positive cortical neurons and was localized mainly in the cytoplasm (Figure 2A). The fluorescent signal from the neurons increased when 5-LOX expression was inhibited by intragastric administration of Zileuton. Western blotting confirmed that 5-LOX expression was significantly reduced by Zileuton as compared to animals in the SAH group. However, inhibition of the PI3K/AKT pathway through intracerebroventricular infusion of LY294002 abolished the Zileuton-induced inhibition of 5-LOX (Figure 2B, 2C). In addition, the levels of phosphorylated AKT (p-AKT) and the P-AKT/AKT ratio was higher in SAH + Zileuton group compared to SAH group (Figure 2D, 2E). However, the beneficial effects of Zileuton were attenuated by LY294002 in SAH + Zileuton + LY294002 group. These findings suggest that Zileuton downregulates 5-LOX overexpression through activation of the PI3K/AKT signaling pathway.

### Role of zileuton in inflammatory action

**Figure 3:**
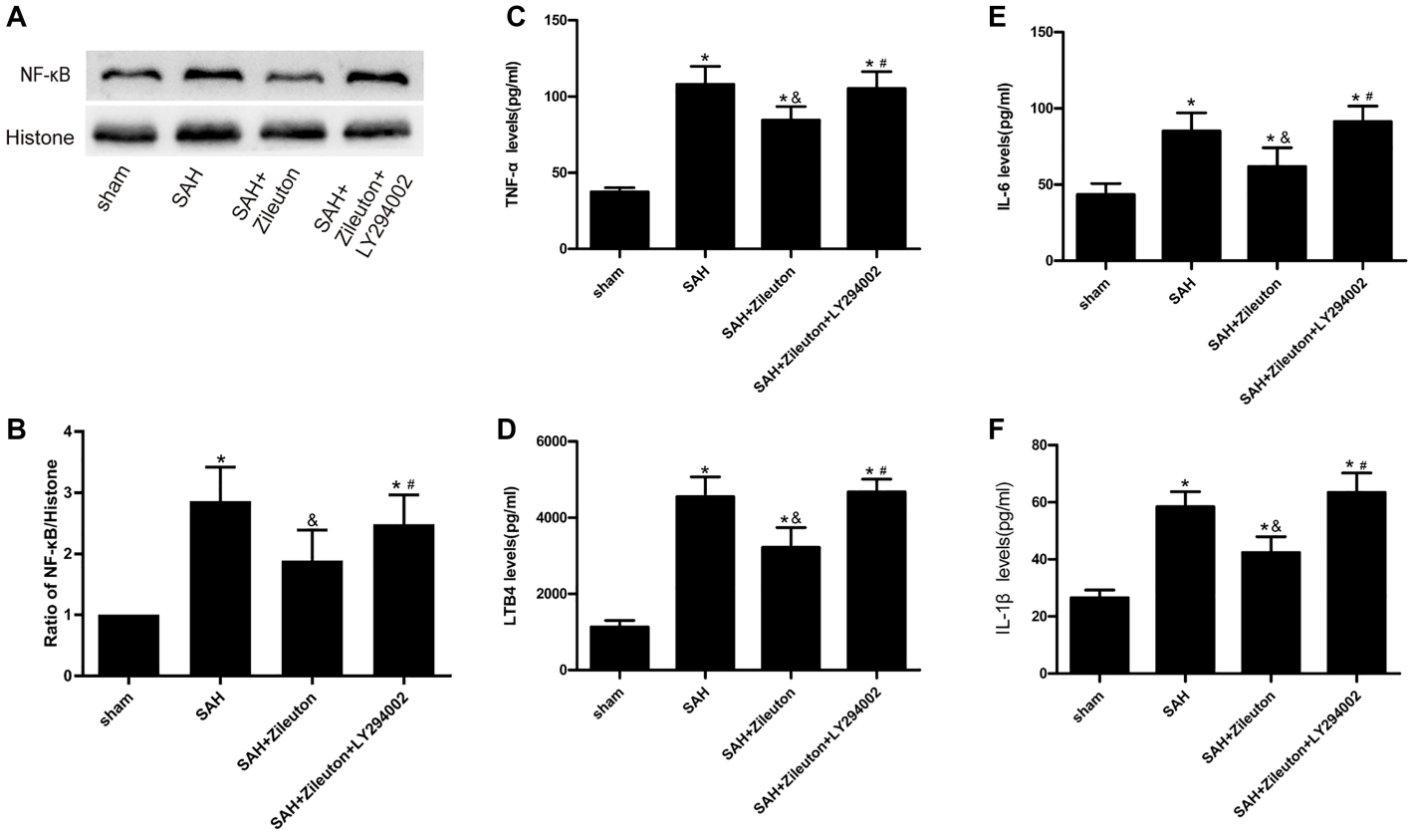
**Zileuton suppresses expression of NF-κB, LTB4, TNF-α, IL-1β and IL-6 after SAH.** (**A**, **B**) Representative images and the quantitative analysis of NF-κB after SAH in rats treat with Zileuton with or without LY294002 (*n* = 6 per group). (**C**–**F**) Effect of Zileuton with or without LY29400 on expression of LTB4, TNF-α, IL-1β and IL-6 after SAH (*n* = 6 in each group). ^*^P < 0.05 vs sham group and ^&^*P* < 0.05 vs SAH group, ^#^*P* < 0.05 vs SAH + Zileuton group.

To explore the effect of Zileuton on the inflammatory response after SAH in rats. Firstly, we used Western blot analyses NF-κB expression. As shown in Figure 3, compared to sham group, the expression of NF-κB was increased after SAH, we also found Zileuton significantly reduced NF-κB levels in SAH + Zileuton group (*P* < 0.01) (Figure 3A, 3B). This effect of Zileuton was again attenuated by LY294002 in the SAH + Zileuton + LY294002 group. In addition, ELISAs showed that serum levels of leukotriene B4 (LTB4) and inflammatory cytokines (TNF-α, IL-6 and IL-1β) were all elevated in SAH group than the sham group (Figure 3C, 3D, 3E, 3F). Zileuton reduced the levels of inflammatory cytokines, and those effects were inhibited by LY29400 (Figure 3C, 3D, 3E, 3F). These results demonstrate that the anti-inflammatory effects of Zileuton are mediated, at least in part, through activation of the PI3K/AKT signaling pathway.

### Zileuton inhibits apoptosis by PI3K/AKT signaling

**Figure 4:**
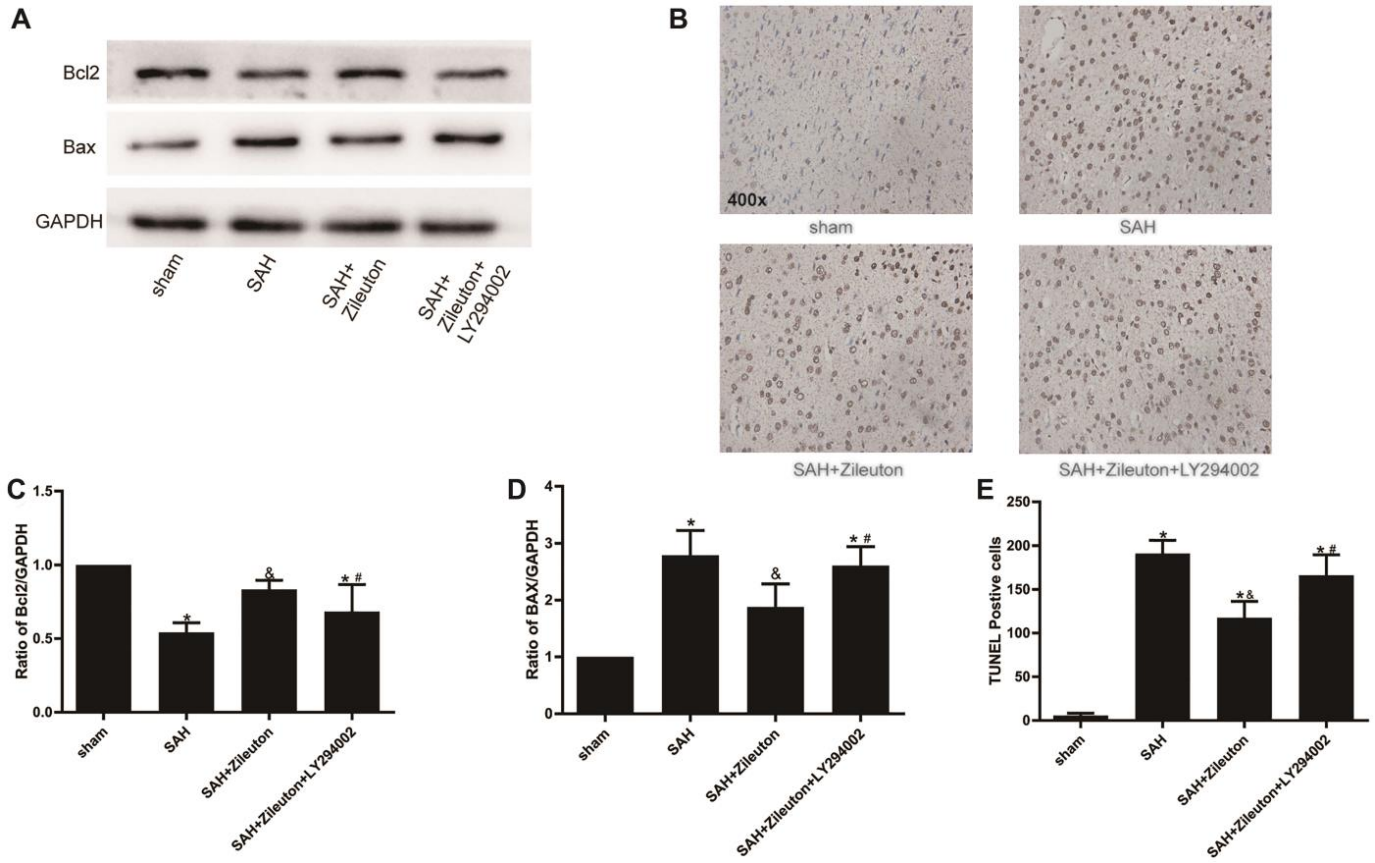
**Zileuton suppresses neuronal apoptosis after SAH.** (**A**, **C**, **D**) Effect of Zileuton with and without LY294002 on expression levels of pro-apoptotic Bax and anti-apoptotic Bcl-2 (*n* = 6 per group). ^*^P < 0.05 vs sham group and ^&^*P* < 0.05 vs SAH group, ^#^*P* < 0.05 vs SAH + Zileuton group. (**B**, **E**) TUNEL analysis of the effect of Zileuton with and without LY294002 on the incidence of apoptosis among neurons after SAH. Original magnification, 400×, *n* = 6 per group. ^*^P < 0.05 vs sham group and ^&^*P* < 0.05 vs SAH group, ^#^*P* < 0.05 vs SAH + Zileuton group.

When we used Western blotting to evaluate the effect of Zileuton on changes in the expression of apoptotic proteins after SAH, we found Zileuton-induced decreases in 5-LOX expression were accompanied by the expression of Bax decreased and Bcl-2 increased (Figure 4A, 4C, 4D). Those effects were reversed by administration of LY294002 (Figure 4A, 4C, 4D). Moreover, TUNEL staining showed that the incidence of apoptosis was reduced by Zileuton, and that effect, too, was reversed by LY294002 (Figure 4B, 4E). These results indicate that Zileuton exerts anti-apoptotic effects after SAH through activation of the PI3K/AKT signaling pathway.

### Zileuton decreases cerebral edema, prevent BBB disruption and improves neurological function

**Figure 5:**
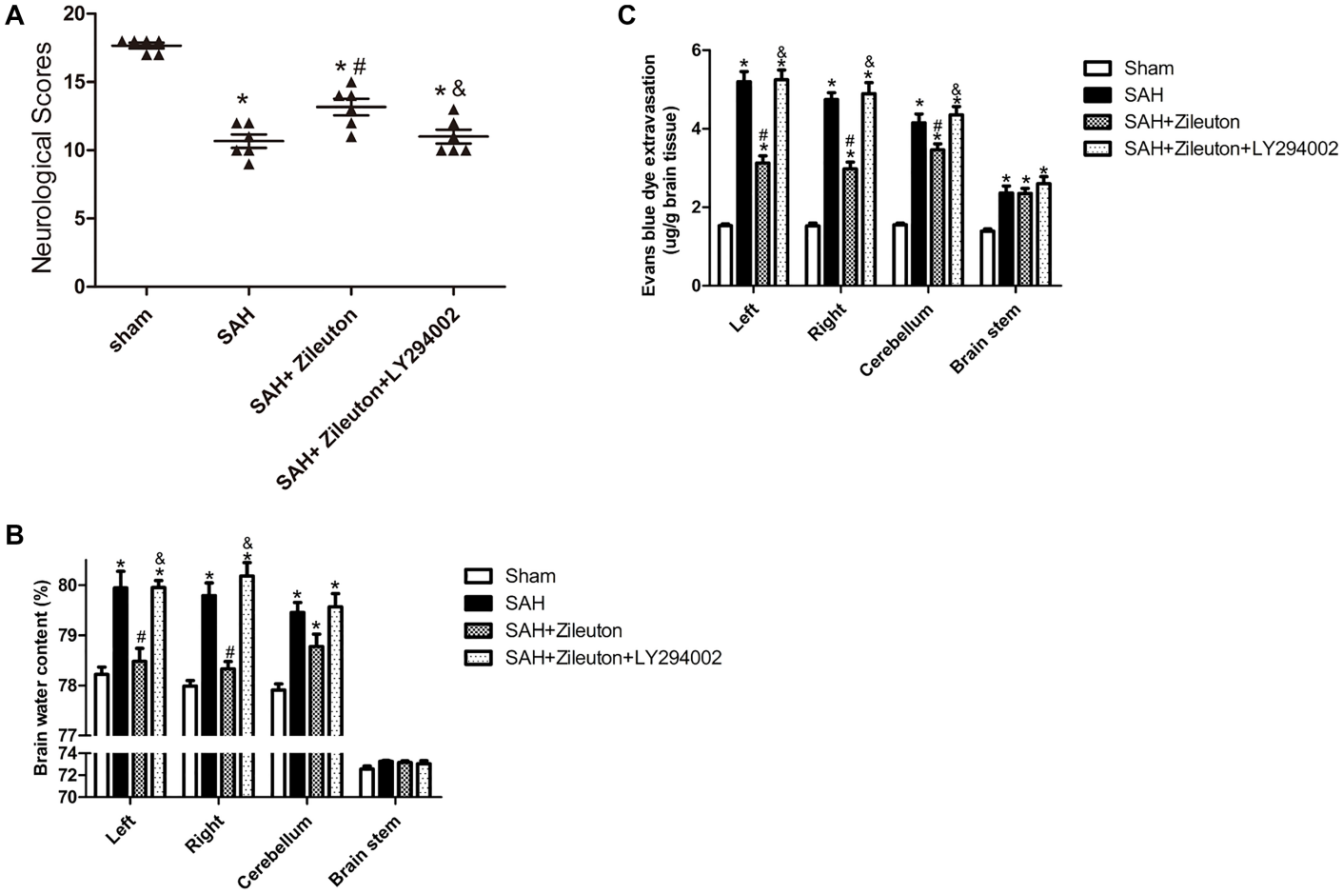
**Zileuton improves neurological scores, BBB permeability and brain water content after SAH**. (**A**–**C**) Effect of Zileuton with and without LY294002 on the indicated neurological functions (**A**); water content in the left and right cortical hemispheres (LH and RH, respectively), brain stem (BS) and cerebellum (CB) (**B**); and BBB permeability in the same brain regions, as indicated by Evans blue extravasation (**C**) (*n* = 6 in each group). ^*^P < 0.05 vs sham group and ^&^*P* < 0.05 vs SAH group, ^#^*P* < 0.05 vs SAH + Zileuton group.

Neurological scores, Brain water content and Evans blue dye extravasation were measured at 24 h following SAH. SAH significantly reduced neurological scores. However, the scores could be significantly increased by Zileuton (Figure 5A). Those effects were accompanied by decreases in brain water content (Figure 5B) and BBB permeability (Figure 5C). All of the beneficial effects of Zileuton were suppressed by LY294002 (Figure 5A, 5B, 5C).

## DISCUSSION

In the present study, we proved that pharmacological inhibition of 5-LOX significantly attenuated EBI and potentially improved the prognosis after SAH. Our results showed that these protective effects were associated with inhibition of pro-inflammatory and pro-apoptotic signaling pathways. 5-LOX colocalized with cortical neurons, and its expression was elevated in brain tissues after SAH. Zileuton significantly reduced levels of 5-LOX, as well as those of various inflammatory and apoptotic mediators, at least in part through activation of the PI3K/AKT signaling pathway. In conclusion, our results demonstrate that 5-LOX plays a crucial role in SAH and that Zileuton may be an effective agent for the treatment of EBI after SAH.

Recent studies have been confirmed that inflammation is a key pathologic manifestation of EBI. The inflammation is derived from expression of inflammatory cytokines and inflammatory mediators induced by SAH and is a key factor contributing to an poor prognosis after SAH [11–13]. Ultimately, the inflammatory response leads to apoptosis, which peaked 24 h after SAH and mainly affected neurons in our model. This inflammatory response is thought to be causatively related to EBI [14]. Our finding that Zileuton suppresses both the inflammatory and apoptotic responses in our model is consistent with earlier studies showing that anti-inflammation and anti-apoptosis treatments had beneficial effects after SAH [11, 15, 16].

5-LOX is a key enzyme in the biosynthesis of leukotrienes and is known to be involved in several inflammatory diseases, including ischemic stroke [5, 17]. Previous studies have shown that expression of 5-LOX in the brain is markedly increased following brain injury caused by trauma or ischemic disease [18–20] and that it mediates the pro-inflammatory and pro-apoptotic responses seen in ischemic disease [6, 21]. Whether or not 5-LOX also involved in the effect of SAH is unclear. However, our findings suggest that expression 5-LOX protein within cortical neurons is significantly increased, which is consistent with 5-LOX participating in EBI after SAH.

Zileuton is an orally administered, selective 5-LOX inhibitor shown to be efficacious and well tolerated in asthma patients [22]. Earlier studies have shown that after ischemic stroke, Zileuton suppresses NF-κB activation and the levels of inflammatory cytokines were decreased (LTB4, TNF-α, IL-6, IL-1β) [19, 21, 23]. 5-LOX catalyzes the synthesis of LTB4 from arachidonic acid [24]. LTB4 then goes on to induce overexpression of TNF-α and IL-6, thereby contributing to the development of an inflammatory environment in ailments such as atherosclerosis [25]. Studies have also demonstrated that NF-κB expression is increased after SAH, and those increases correlate with 5-LOX expression. In this study, we detected the levels of inflammatory cytokines by ELISA, we found that all of them increased after SAH, the levels were inhibited by treatment with Zileuton. Moreover, the Zileuton-induced reductions in 5-LOX and LTB4 were associated with a decrease in NF-ĸB activity after SAH. These results provide further evidence supporting the idea that by inducing inflammatory responses, 5-LOX plays a causative role in EBI after SAH.

Earlier studies showed that Zileuton reduces apoptosis and relieves inflammatory events in various models [6, 26, 27]. Consistent with those findings, we observed that Zileuton suppressed pro-apoptotic transcriptional changes (increased expression of BAX and decreased expression of Bcl2) induced by SAH as well as the corresponding increase in the incidence of apoptotic (TUNEL-positive) cells. Several recent studies have shown that anti-inflammatory and anti-apoptotic signaling after SAH is mediated via the PI3K/AKT pathway [8, 28, 29] and that PI3K/Akt are major upstream elements regulating NF-κB pathway [30]. In the present study, we found that Zileuton significantly upregulated levels of activated (phosphorylated) Akt after SAH while decreasing NF-κB, LTB4, TNF-α, IL-6 and IL-1β. Activated Akt inhibits pro-apoptosis proteins such as Bax and promotes anti-apoptotic proteins such as Bcl-2 [31]. At the same time, Zileuton could reduced cerebral edema and prevented BBB disruption and attenuated the neurological deficits. However, these beneficial effects of Zileuton have been reversed by the PI3K-specific inhibitor LY294002. These results indicate that Zileuton inhibit inflammatory and apoptotic factors after SAH through PI3k/Akt signaling pathway.

In summary, our present study shows that 5-LOX contributes to EBI in a rat model of SAH by mediating inflammation and neuronal apoptosis. Inhibition of 5-LOX by Zileuton alleviates the SAH-induced inflammation and apoptosis through PI3K/AKT signaling pathway, which suggests 5-LOX inhibition may represent a potentially effective approach to SAH treatment. These data suggest that 5-LOX is a promising therapeutic agent for SAH treatment.

## MATERIALS AND METHODS

### Ethical standards

Rats in this study were raised and cared for in the Laboratory Animal Resource Center (LARC) at Chongqing Medical University. All experiments were performed following the approval of the Ethical Committee of Chongqing Medical University and according to the Guide for Care and Use of Laboratory Animals (US National Institutes of Health). This study adhered to all ARRIVE (Animal Research: Reporting *in vivo* Experiments) guidelines.

### Animals and SAH model

Male Sprague-Dawley (SD) rats (280 to 330 g) were randomly divided into different groups: sham, SAH, SAH + Zileuton, SAH + Zileuton + LY294002. The endovascular perforation model of SAH in rats was performed as reported previously [32]. In brief, rats were anesthetized using an intraperitoneal injection of sodium pentobarbital (50mg/kg). Blunt dissection of internal carotid artery (ICA) and external carotid artery (ECA) under microscope, a sharpened 4-0 monofilament nylon suture was inserted into the right internal carotid artery (ICA) from external carotid artery (ECA), perforating the bifurcation of the anterior and middle cerebral arteries. Rats in sham group underwent the same operation without perforation, which were then sacrificed at the indicated times during subsequent experiments. In this study, the surgical operations and data analysis were performed by a researcher blinded to the animal treatment group.

### Drug administration

Firstly, Zileuton 50 mg/kg (TargetMol, Boston, MA) was dissolved in DMSO, further dissolved in normal saline to 0.5% DMSO. Rats were administered the drug by intragastric infusion after SAH. LY294002 (Selleck, USA) was dissolved in DMSO and then diluted in PBS (10 μM). LY294002 was infused intracerebroventricularly beginning 1.5 h after induction of SAH [13]. Rats were treated with the same volume of DMSO as the vehicle control and time point as Zileuton.

### Western blot analysis

Brain tissues were collected at different time points after SAH induction , Western blot was performed as previously described [12]. Primary antibodies used included 5-LOX (1:5000, Abcam), p-AKT (1:5000, Abcam), AKT (1:5000, Abcam), Bax (1:5000, Abcam), GAPDH (1:5000, Abcam), NF-κB (1:1000, Immunoway), Bcl2 (1:1000, Immunoway), and histone (1:1000, Proteintech). Blots were detected by enhanced chemiluminescence and quantified using Quantity One software. GAPDH and Histone were used as loading controls for whole cell and nuclear proteins.

### Immunofluorescence

Double-fluorescent staining was performed 24 h after SAH as described previously [33]. Ten-micrometer-thick sections of SAH rat brains were incubated with rabbit anti-5-LOX antibody (1:50, Abcam) and mouse monoclonal anti-NeuN (1:100, Monoclonal) overnight at 4°C. Alexa Fluor 488-conjugated donkey anti-mouse IgG (H + L) (1:200, Proteintech) and Alexa Fluor Cy3-conjugated goat anti-rabbit IgG (H + L) (1:200, Proteintech) were used for fluorescent secondary antibodies. The same staining procedures were conducted without primary antibodies for the negative controls. Fluorescence microscopy (Nikon A1 + R, Japan) was used to detect 5-LOX localization in neurons.

### TUNEL

TUNEL was implemented according to the manufacturer’s protocol, as described previously [34]. For each animal, the numbers of TUNEL-positive cells in five separate high-magnification (400×) fields in the ipsilateral basal cortex (left) were counted using an Olympus microscope by observers blinded the treatment conditions. The number of TUNEL-positive cells per square millimeter was indicates the severity of the brain injury. A labelling solution without TUNEL reagent was used as Negative controls.

### ELISA analysis

The levels of inflammatory cytokines (LTB4, TNF-α, IL-1β and IL-6) were measured by ELISA Kits (Wuhan Colorful Gene Biological Technology, Wuhan, China). Assays were performed according to the manufacturer's instructions.

### Neurobehavioral score

Neurological scores were conducted at 24 h after SAH, which using the scoring method reported by Sugawara [35]. The scores were evaluated by an observer who was blinded to each group. A lower score indicated serious neurological deficits.

### Brain water content

Under deep anesthesia, at 24 h after SAH, the brains of each group were quickly divided into the left and right hemisphere, cerebellum, and brain stem. These portions were immediately weighed (wet weight) and then dried in the oven for 72 h at 105°C (dry weight). The brain water content percentage was calculated using the following equation: (wet weight to dry weight)/wet weight × 100% [13].

### BBB disruption

The permeability of the BBB was evaluated based on Evans blue extravasation, as previously described [36]. The brain level of Evans blue was determined at 615 nm for spectrophotometric quantification.

### Statistical analysis

All the data in this study were expressed as the mean ± SEM. Firstly, the Shapiro-Wilk normality test was used to confirm data normality. After passed the normality test, data were analyzed by the homogeneity test of variances. Once equal the variance assumption were confirmed, significant differences between all groups were analyzed using one-way ANOVA followed by Tukey’s multiple comparison post hoc analysis. Values of *p* < 0.05 were considered statistically significant. All statistical analyses were performed using GraphPad Prism for Windows.
